# Perspectives of women participating in a cervical cancer screening campaign with community-based HPV self-sampling in rural western Kenya: a qualitative study

**DOI:** 10.1186/s12905-019-0778-2

**Published:** 2019-06-13

**Authors:** Sandra Y. Oketch, Zachary Kwena, Yujung Choi, Konyin Adewumi, Michelle Moghadassi, Elizabeth A. Bukusi, Megan J. Huchko

**Affiliations:** 1 Center for Microbiology Research, Kenya Medical Research Institute, P. O. Box 54840 00200, Mbagathi Road, Nairobi, Kenya; 2 Duke Global Health Institute, Box 90519, 310 Trent Drive, Durham, NC 27710 USA; 3 Department of Obstetrics and Gynecology, University of California San Francisco, 550 16th Street, 3749, San Francisco, CA 94158 USA; 4 Department of Obstetrics and Gynecology, University of Nairobi, P. O. Box 54840 00200, Nairobi, Kenya; 5 Department of Obstetrics and Gynecology, Aga Khan University, P. O. Box 30270 00100, Third Avenue, Limuru Rd, Nairobi, Kenya; 6 Departments of Obstetrics and Gynecology, University of Washington, P. O. Box 356460, Seattle, WA 98195 USA; 7 Department of Obstetrics and Gynecology, Duke University, Box 90519, 310 Trent Drive, Durham, NC 27710 USA

**Keywords:** HPV self-sampling, Cervical cancer screening, COM-B, Theoretical domains framework, LMIC

## Abstract

**Background:**

Despite cervical cancer being preventable with effective screening programs, it is the most common cancer and the leading cause of cancer-related death among women in many countries in Africa. Screening involving pelvic examination may not be feasible or acceptable in limited-resource settings. We sought to evaluate women’s perspectives on human papillomavirus (HPV) self-sampling as part of a larger trial on cervical cancer prevention implementation strategies in rural western Kenya.

**Methods:**

We invited 120 women participating in a cluster randomized trial of cervical cancer screening implementation strategies in Migori County, Kenya for in-depth interviews. We explored reasons for testing, experience with and ability to complete HPV self-sampling, importance of clinician involvement during screening, factors and people contributing to screening decision-making, and ways to encourage other women to come for screening. We used validated theoretical frameworks to analyze the qualitative data.

**Results:**

Women reported having positive experiences with the HPV self-sampling strategy. The factors facilitating uptake included knowledge and beliefs such as prior awareness of HPV, personal perception of cervical cancer risk, desire for improved health outcomes, and peer and partner encouragement. Logistical and screening facilitators included confidence in the ability to complete HPV self-sampling strategy, proximity to screening sites and feelings of privacy and comfort conducting the HPV self- sampling. The barriers to screening included fear of need for a pelvic exam, fear of disease and death associated with cervical cancer. We classified these findings as capabilities, opportunities and motivations for health behavior using the COM-B framework.

**Conclusions:**

Overall, HPV self-sampling was an acceptable cervical cancer screening strategy that seemed to meet the needs of the women in this community. These findings will further inform aspects of implementation, including outreach messaging, health education, screening sites and emphasis on availability and effectiveness of preventative treatment for women who screen positive.

## Background

Cervical cancer deaths worldwide were estimated at 275,000 annually and over 530,000 new cases diagnosed [1]. This is despite the fact that cervical cancer is preventable with effective screening and treatment of pre-invasive lesions [2]. The incidence of cervical cancer is highest in low- and middle-income countries (LMICs) where about 80% of new cases [3] and 85% of deaths occur [4]. It is the most common cancer affecting women and the leading cause of cancer deaths in many countries in Africa [5, 6]. In Kenya, cervical cancer is the leading female cancer and cause of cancer-related deaths for women with an incidence of 20.5 per 100,000 women [5]. New cases and mortality are expected to rise by 75% by 2025 in the absence of substantial scale interventions for screening and early treatment [7].

While high quality, accessible and acceptable screening programs are necessary to prevent cervical cancer [8], trained human resources, functional referral and laboratory facilities, and lack of transport and tracking systems make cytology-based screening strategies unfeasible in most LMICs [2]. Women in LMICs have a low screening rate with only 3.5% of women aged between 25 to 64 years screened in any 3-year period [5]. High-risk human papillomavirus (HPV) is the primary cause of almost all cervical cancer [7]. Testing for HPV is a simple, low-cost technology that has been shown to reduce the cervical cancer incidence and related mortality when directly coupled with outpatient cryotherapy treatment for women testing HPV positive [2, 9, 10]. The World Health Organization (WHO) recommends a simplified HPV screen & treat as a strategy for cervical cancer control in LMICs [11]. To increase screening coverage, HPV testing has been offered to women via self-collection, removing the need for a pelvic exam, clinic setting and a trained provider [12–17].

Although the strategy of HPV self- sampling would address many logistical barriers, the ultimate success of this strategy depends upon its acceptability among women in the target population. Studies have confirmed a high acceptability to HPV self-sampling in different settings [15, 18, 19]. Though other studies have compared HPV collection through clinician versus self-sampling [18] only a few studies have provided self-sampling in community settings [15, 19] thus, the need to investigate whether this strategy would be an acceptable form of screening. This has not been studied in rural western Kenya, a low-resource setting with limited access to health facilities and multiple barriers to pelvic exams that could be avoided in a community based self-sampling strategy [20]. We sought to examine women’s perspective and experience with HPV self-sampling using two frameworks that merges internal, interpersonal and systems factors, of the theoretical domain frameworks (TDF) mapped to the capacity (C), opportunity (O) and motivation (M) to bring about behavior (B) change (COM-B) [21, 22].

## Methods

### Design

This qualitative study was conducted as part of a larger cluster-randomized trial comparing two implementation strategies for community-based HPV testing carried out between January and September 2016 in Migori County, Kenya (Clinicaltrials.gov, NCT02124252) [23]. We sought to determine women’s perspectives on acceptability of and experiences with an HPV self-sampling strategy through in-depth interviews (IDIs) with a purposive sample of 120 women participating in the community driven cervical cancer prevention program.

### Parent study description and settings

Migori County is located in Western Kenya, with some areas bordering Lake Victoria. This area has one of the highest HIV prevalence rates in the region (15.1%) [24, 25]. The parent study enrolled women aged 25 to 65 years living within selected communities within Migori County, and offered HPV-based self- screening in health facilities or through community health campaigns between January to September 2016 [23]. The main outcomes and study design are presented elsewhere [26]. Relevant to this study, Community Health Volunteers (CHVs) explained the self-sampling procedure during outreach and mobilization activities in public, group settings and through door-to-door mobilization. More detailed messages were provided immediately prior to screening through a group educational module in both health facility and community settings. Posters with diagrams and instructions for self-collection were displayed in private rooms at campaign sites and health facilities where the self-sampling took place. Specimens were collected using careHPV™ (Qiagen, Gaithersburg, MD) in a private area and returned to the CHVs, who periodically transported them to the laboratory in the Migori County Referral Hospital for processing. Participants received their results in approximately two weeks via their choice of text message, phone call, or home visit.

### Sample size and sampling framework

For this qualitative study, we used purposive sampling and asked CHVs to identify participants who would be willing to share their experiences with screening. We determined that a sample size of 10 interviews per community would provide enough information and variation in data within the target population [27]. We therefore recruited participants until we reached ten participants from each of the twelve study communities. The face-to-face interviews were conducted by trained qualitative interviewers after women underwent the self-sampling at either the community health campaign or health facility.

The questions from the IDI guide were developed from literature review and had both multiple choice and open-ended sections. Quantitative measures included questions on socio-demographic attributes and acceptability of HPV self-sampling. They were followed by open-ended questions that explored reasons for testing, self-sampling experience, importance of clinician involvement during screening, the factors and people contributing to screening decision, and what can be done to encourage other women in the community to come for screening. The guide was developed in English, translated to the local languages (Luo and Kiswahili), and then back-translated into English to ensure accuracy. IDIs lasted approximately 20–30 min and were audio-recorded into an MP3 file and then transcribed and translated. All translations were read again along with the audio by a member of the study team to confirm accuracy.

#### Data analysis

We utilized a theoretical thematic analysis approach to identify themes during the coding process [28]. A codebook was developed based on a thorough review of five sample IDI transcripts. Using NVivo10 [29], a group of five members of the research team coded the transcripts independently, with each transcript coded by two members producing coding reports for joint review. Coding reports were discussed in a series of meetings to refine the codebook and guide the analysis and the themes considered in relation to the domains in the TDF and then mapped onto the segments of COM-B framework.

Theoretical Domains Framework is used to identify the facilitators and barriers and influences on behavior in application of specific evidence based behaviors and can be used in qualitative studies to understand implementation and provide insight to an intervention and its mechanism of change [21, 30–32]. The TDF domains have been shown to have influence on the behaviors across all levels of health implementation and uptake [21, 30]. The sources of behavior, COM-B, has been used to understand behavior change interventions and it categorizes behavior in terms of the three key determinants of capability, opportunity and motivation [33] . The TDF has been validated for use in the COM-B, behavior change wheel and the two frameworks are used together to inform intervention and describe behavior change in implementation and behavior change research [21, 22]. The combination of these two frameworks guided our analysis in an attempt to ensure an understanding of women’s perspective on HPV self-sampling to assess the influence of beliefs and behaviors to inform of this and future implementation strategy.

## Results

The TDF allowed for the identification of the facilitators to screening which included awareness and acceptability of HPV self-sampling, personal perception of cervical cancer risk, confidence in the ability to complete HPV self-sampling, peer and partner encouragement, privacy and comfort conducting the HPV self-sampling, proximity to screening sites, improved health outcomes and desire to know HPV status. The identified barriers to screening were: social stigma associated with cervical cancer, a number of fears were reflected which included fear of pelvic exam and fear of disease and death. These domains were mapped to the behavior change constructs of capability, opportunity and motivation (Table 1).

**Table 1:** Key findings from women’s perspective on HPV sampling mapped onto the TDF and COM-B frameworks

COM-B Component	TDF Domain	TDF Domain Description	Key Findings
Capability	Knowledge	An awareness of the existence of something	Awareness and acceptability of HPV self-sampling
	Skills	An ability or proficiency acquired through practice	Confidence in their ability to complete HPV self-sampling strategy Role of clinicians in HPV sample collection
Opportunity	Social Influences	Those interpersonal processes that can cause an individual to change their thoughts, feelings, or behaviors	Peer and partner encouragement Social stigma associated with cervical cancer
	Environmental Context and Resources	Any circumstance of a person’s situation or environment that discourages or encourages the development of skills and abilities, independence, social competence, and adaptive behavior	Proximity to cervical cancer screening sites Fear of pelvic screening
Motivation	Social Role and Identity	A coherent set of behaviors and displayed personal qualities of an individual in a social or work setting	Feeling of privacy and comfort conducting the HPV self-sampling
	Beliefs about Capabilities	Acceptance of the truth, reality, or validity about an ability, talent, or facility that a person can put to constructive use	Personal perception of cervical cancer risk Screening will improve health outcomes
	Beliefs about Consequences	Acceptance of the truth, reality, or validity about outcomes of a behavior in a given situation	Fear of disease and death

Note: Description data from Cane et al. [23]

The mean age of the 120 women interviewed was 36.1 years, (SD = 9.55). Over half (59.4%) the women interviewed had up to a primary school education. Over three quarters (78%) were married with a median of 4 (IQR 3–5) children, and 16% of women had undergone prior screening (Table 2).

**Table 2:** Socio-demographic characteristics and reproductive history of participants (*n* = 117)

Socio-demographic Characteristics	n (%)	Reproductive History Characteristics	n (%)
Age Mean (SD)	36.08 (9.55)	Number of children (IQR)	4 (3,5)
Education Level		Family planning use (*n* = 80)	
Primary	77 (55.8)	Yes	36 (45.0)
Secondary	28 (24.0)	No	44 (55.0)
College and beyond	8 (6.8)		
None	4 (3.4)		
Occupation		Previous cervical cancer screening (n = 80)	
Agriculture and fishing	42 (35.9)	Yes	16 (20.0)
Sales and services	32 (27.3)	No	64 (80.0)
Professional/Technical/Managerial	16 (13.7)	Previous cervical cancer screening type	
Skilled and unskilled manual	11 (9.4)	Pap smear	6 (37.5)
Housewife/None	16 (13.7)	VIA/VILI	10 (62.5)
Relationship Status		Previous cervical cancer screening result	
Married/Partnered	91 (77.8)	Negative	15 (93.8)
Widowed/Divorced	24 (20.5)	Positive	1 (6.2)
Single	2 (1.7)		

Note: Missing quantitative data for 3 participants

### Capability

Themes related to capability nested within the TDF domains of knowledge and skills and included awareness and acceptability of HPV self-sampling and participant’s confidence in their ability to complete HPV self-sampling strategy. There were all seen to influence their decision to be screened.

#### Awareness and acceptability of HPV self-sampling

The majority of women mentioned both their own volition and an awareness of self-sampling availability in screening decision making. One married woman with no prior screening stated “*It was my own decision when I heard about it I saw it wise to go for it*”. Most women thought that the self-sampling would be widely accepted by the community “*because they are afraid of the metal insertion procedure. They will go for this*”. Some women had anxiety about the procedure, seen in the following quotes: “*Those who haven’t gone for the test feel that the procedure can lead into a scratch* …: ” and that “*… .some women told me that they are scared of having to use the care brush”.* Others felt that the privacy allowed with self-sampling afforded them agency.

*“They will [get screened] because the test is not painful and it is not difficult. If you go to the testing room, you are all alone just like in your own room, so you are just free”.* (Married woman with no prior screening)

#### Confidence in the HPV self-sampling strategy

When asked whether women would be able to complete the testing, we had almost uniform responses of confidence. Some of the facilitators to screening completion included “… ..*the procedure does not take time*”, “*because it is private and one performs the test by herself*” and “*… the new type of testing was easy to undertake with no difficulties”.*

#### Role of clinicians in HPV sample collection

Despite women’s confidence in and preference for a self-collection without a pelvic exam, there were diverse opinions on the need for clinician presence. The women who expressed a desire for clinicians felt their role would be to provide any additional information about screening and results, and be available for treatment after a HPV positive screen test. We did not find any women who preferred clinicians to provide screening via pelvic exam.

*“In relation to the new self-test kits that have emerged, it isn’t a must that the health care provider to be next to you … ”.* (Unmarried woman with no prior screening)

*“I would wish for the clinician to be there so that after getting tested and I end up being positive the doctor is able to … [perform the] treatment already but not that I look for money and go to Migori”. (Married woman, with no prior* screening*)*

### Opportunity

Themes related to opportunity nested within the TDF domain of social influences included peer and partner encouragement, social stigma associated with cervical cancer. The environmental resources and context domains were mapped and the ideas about proximity to screening sites and fear of pelvic screening as influencing behavior and attitude to screening uptake.

#### Peer and partner encouragement

Women cited peer and partner encouragement as influential in their decision making process, which shows the significance of these support systems to screening. A number of women recounted how their peers shared their self-sampling experience which encouraged them to get screened. Common sentiments included *“those who have screened encourage those who have not by telling them the benefits of screening …*” and *“I was encouraged by those of us who have been through the process”.* Other women mentioned the role their male spouses played which included encouragement and support to get screened.

*“I had been talking with my husband about it [HPV self-testing] and … … My husband could encourage me that cancer is a deadly disease and that I should go for early screening so that I can be able to be screened”.* (Married woman with no prior screening)

#### Social stigma associated with cervical cancer

There was an indication of stigma where some women feared being associated with having the disease. *“Generally people fear others seeing them going for screening because they might think one has the* disease *…*.” *.* There were also feelings that general belief that cervical cancer is a life threatening disease was a major hindrances to testing. Women suggested education and counseling as a measure to counter the stigma and create positive attitudes towards screening.

*“They will not accept because they feel that cancer is a deadly disease. So I feel that they should be counseled and taught about this disease in such a way that they would feel free to do the cervical cancer test”.* (Unmarried woman with prior screening using VIA/VILI)

#### Proximity to cervical cancer screening sites

A majority of women reported that long distance travel may be a barrier due to high travel costs and travel time away from home and chores. Some suggested that HPV screening was a better option when offered *“at the community site, because you may even lack fare when screening is taken to a far place.”* This same sentiment was expressed about treatment sites and availability.

*“It depends on how I am that way because, if it were near I would perform my responsibilities and later go for treatment but if it is far transport will be an issue, so it’s better if it were near because I will be able to go for the treatment and later perform my duties”.* (Unmarried woman with no prior screening)

Many of the communities are located considerable distances (average 24 km) from the health facilities and most of these facilities lack the screening and treatment resources needed. The inadequacy of the resources and proximity to screening services intensifies the lack of screening and under screening. The majority of women appreciated the fact that the HPV screening services were brought closer to them through community outreach and in the nearby health centers which facilitated their ability to access HPV self-sampling.

*“Maybe if it [HPV screening] could have been far I wouldn’t have gone and so the fact that it [HPV screening] was closer is what made me make the decision to come”.* (Married woman with no prior screening)

#### Fear of pelvic screening

Fear of cervical cancer screening was reflected when women mentioned that they would feel pain from the pelvic exam procedure which acted as a possible barrier to screening. There was also the feeling of embarrassment if male providers offered screening through a pelvic exam. All of these factors from the women’s previous screening experiences made them hesitant to go for screening again, and therefore making the HPV self-sampling approach an attractive opportunity.

*“The fact that I get to do the test on my own and I don’t have to expose myself to a male doctor {laughs} the way it was before.”* (Married woman with no prior screening)

*“ … .I used to hear that those who were being tested were experiencing pain because there was something that was being inserted inside them, it was great pain, and this scared me from going for the test, so when you introduced the new type of testing, I made a decision to come for it”.* (Married woman with no prior screening)

### Motivation

Themes related to motivation nested within the TDF domain of social identity included the feelings of privacy and comfort conducting the HPV self-sampling. The beliefs about capabilities domains identified included personal perception of cervical cancer risk and improved health outcomes. The theme of fear of disease and death was mapped onto the domain of beliefs about consequences.

#### Feeling of privacy and comfort conducting the HPV self- sampling

Most women reiterated that self-sampling eliminated the embarrassment and fear they had from pelvic exam by a clinician and reported having a positive experience conducting the self-sampling compared to previous testing with a pelvic exam. *“Because it is private and one performs the test by herself”,* “*… … and the procedure does not take time”.*

*“What I didn’t like is that initially there was a way through which cancer was being screened [with a pelvic exam] … that I didn’t like so I was scared. I felt that these doctors are from the community and maybe I would meet up with them at a later time in the village and so this was a burden, but with the self-test kit I felt it was good because I could test myself”.* (Unmarried woman with no prior screening)

#### Personal perception of cervical cancer risk

Most of the women were aware of the fact that they were susceptible to cervical cancer and that early HPV screening would prevent disease progression. *“I wanted to know my status, I might be having cancer yet I don’t know, so I wanted to know so that I seek treatment”.* For some HIV-positive women, they wanted to employ any necessary prevention mechanisms in recognition of their increased susceptibility to complications.

*“I wanted to know my [HPV] status so that if at all I test positive I can be helped before it progresses secondly, me knowing my [HPV] status, will be good in a way that I would be able to start on early treatment, even if I don’t have I will be able to know and that is why I decided to come for the test”.* (Married woman with prior screening using Pap smear)

*“First and foremost I am HIV positive so my immune system is low, and so it forces me to always come and do the test”.* (Married woman with prior screening using Pap smear)

#### Screening will improve health outcomes

Women reported that their reason for HPV self-sampling was to improve their health outcome by taking necessary steps based on the test result where those who screen positive for HPV are able to seek early treatment as a preventive measure. Some women stated the role the education played in their understanding of screening in improving their health outcome as illustrated by this quote *“I made the decision because I was taught that if it [HPV] gets [into] and stays in the uterus, it turns to full-blown cancer and it shortens one’s life, and if detected early you can still find help”.* As such, indicating that education and awareness of the cervical cancer prevention initiated and influenced their personal decision to screen.

*“I heard of the benefits of doing the [HPV] screening to prevent it, because prevention is better than cure.”* (Married woman with no prior screening)

*“The reason why we do HPV testing is to make one aware of their status in relation to HPV, such that if they are found to be positive then they would know how to get treatment in good time when it’s not yet too late”.* (Unmarried woman with prior screening using VIA/VILI)

One woman expressed a desire to test for HPV to avoid complications that would interfere with childbearing. This acted as a motivator to seek cervical cancer screening:

*“What contributed to my decision to get screened was I wanted to know whether I was HPV positive or negative, according to my age I still need to have children, so if you keep silent about it without knowing what is going on, it is good if you know earlier”.* (Married woman with no prior screening)

#### Fear of disease and death

Some women mentioned fear of disease and death if they do not get early screening, so this encouraged women to get screened to know their HPV test results so as to be able to seek treatment in time as late detection and failure to seek treatment was associated with death.

“*I have seen cervical cancer kill those who did not want to go to the hospital for screening. That is why when I heard about it, I decided to go for the screening so that in case I am HPV positive, I find help”.* (Unmarried woman with no prior screening)

*“You know some fear. One might say, I will go there and be found to have the virus … . That is what scares a lot of people. They really do not know what they are going to screen for. They wait until they get worse that is when they go”.* (Married woman with prior screening using VIA/VILI)

## Discussion

Innovative cervical cancer screening strategies are an essential part of the prevention cascade that will ultimately reduce the global disparity in cervical cancer incidence. Effective implementation of cervical cancer prevention services requires reaching women at the community level with programs that are acceptable, inexpensive, easy to use, and sustainable. HPV self-sampling may bridge the gap for women in underserved areas as it has the potential to increase screening availability in more remote areas, by using a less invasive procedure. We found that women in rural western Kenya had positive expectations of and personal experiences with HPV self-sampling, which compared favorably to prior experience with or beliefs about pelvic exams which is consistent to other studies done in Africa and Asia [18, 19, 33–35]. Also, HPV self-sampling has been found to improve screening uptake [36]. Acceptability of this method suggests an opportunity to increase attendance and screening through community based programs in LMICs.

Using the TDF to map drivers of behavior change (COM-B), we found that environmental context and resources were the most widely cited, followed by social influences, both under the opportunity construct. Women reported that cervical cancer screening using an exam-based method elicited a number of barriers that were not part of HPV self-screening. The findings on pelvic exam as a barrier to screening is consistent with other studies done in Africa and Asia [18, 19, 33–35] which showed concerns around speculum use, embarrassment, and reluctance to have a male provider perform the exam [37–39]. Consequently, employing the self-sampling approach provides the opportunity to eliminate some of these barriers and improve on screening uptake. Health system factors such as access and proximity to care were also identified as barriers to conventional screening, as they have also acted for other prevention programs as has been found in other studies [37, 38, 40–43]. Important facets of environmental context and resources include the need to increase community outreach, provide access to screening and treatment services in central locations and ensure privacy for women undergoing counseling and testing.

We also identified some areas of stigma around HPV and cervical cancer. While this was not a frequent finding, it concurs with other studies which have also found that having pre cancer or cervical cancer has been stigmatized with women fearing being “talked” about or facing discrimination [37, 39]. Our results support the need for improved community education and stigma reduction to ensure high uptake of screening and support for women who screen positive. We found that peer and partner encouragement, mapped to the social influences domain, were common and an important motivator for screening. The social support systems of peer and partner support is important in successful implementation of health prevention programs [40] consequently, ensure high cervical screening participation and attendance.

The motivation construct illustrated the women’s behavior to screening under the beliefs about capabilities domain whereby the perception of one’s susceptibility to cervical cancer does affect screening behavior and for women who expressed personal susceptibility to cervical cancer believed it was necessary to have HPV self- test done and vice versa to those who expressed lack of personal susceptibility [44–46]. As has been previously documented, knowledge and perception play a key role in influencing screening behavior [47–51]. The domain on belief about consequences also reflected the women’s motivation construct that highlight barriers to HPV screening which include the fear of disease and death which has been identified in other studies [18, 52]. To address the issue of fear of potentially learning that one has cancer, a rigorous screening awareness campaign is needed to empower the women and address the fears of screening and an understanding to personal perception of cervical cancer risk.

This study is limited by the inclusion of data only from women who had already self-sampled for HPV. As such, we do not have the perspectives of those who did not participate, a population who may be the most difficult to reach and may better articulate the barriers to self-sampling. We also sampled a population with a high HIV prevalence and a strong response to the epidemic, which may consequently have been exposed to prior research studies and health interventions that may have influenced their health beliefs and behaviors. These may impact their response towards the HPV self-sampling uptake and thus may also not be generalizable to other rural populations without such a robust response to HIV. These limitations notwithstanding, our results inform of women’s experiences and insights to HPV self- sampling approaches to this settings. Also, due to the study’s cluster randomized design, it provides a representative sample and the participants were potentially comfortable due to the fact that the interviews were conducted in their native languages. This study’s use of behavior change theory provides a novel aspect of understanding women’s perspectives and experiences that influences their screening behavior.

## Conclusion

A combination of the TDF and COM-B allowed for identification of the women’s facilitators and barriers to screening which in turn described potential drivers of behavior change in the relation to screening with self-collected HPV. The health systems in low income regions have limited financial and human resources therefore prevention of cervical cancer remains an unmet priority. Thus, acceptability of self-sampling in the community setting is encouraging as these can ensure focus on treatment efforts for those who screen positive. Increased community awareness and emphasis that screening would not involve a pelvic exam, can be done in privacy, and would still involve provider counseling and may encourage even broader participation in organized screening campaigns. Cervical cancer screening access in terms of distance and convenience provide opportunities to increase screening uptake. Future research efforts should focus on the self-sampling delivery and infrastructure strengthening.

## Data Availability

The data sets used and/or analysed during the current study are available from the corresponding author upon reasonable request.

## Abbreviations

CHVs: Community Health Volunteers
COM- B: Capability, Opportunity and Motivation in Behavior
HIV: Human Immunodeficiency Virus
HPV: Human papilloma Virus
IDI: In depth interview
LMICs: Low and Middle Income Countries
TDF: Theoretical Domain Framework
VIA: Visual inspection with acetic acid
VILI: Visual inspection with lugols iodine solution
WHO: World Health Organization

## References

[1] de Sanjosé S, Serrano B, Castellsagué X, Brotons M, Muñoz J, Bruni L, Bosch FX (2012). Human papillomavirus (HPV) and related cancers in the global Alliance for vaccines and immunization (GAVI) countries: a WHO/ICO HPV information Centre report. Vaccine.

[2] Denny L, Kuhn L, De Souza M, Pollack AE, Dupree W, Wright TC (2005). Screen-and-treat approaches for cervical cancer prevention in low-resource settings: a randomized controlled trial. J Am Med Assoc.

[3] Sherris J, Herdman C, Elias C (2001). Beyond our Borders: cervical cancer in the developing world. West J Med.

[4] Ferlay J, Shin H-R, Bray F, Foreman D, Mathers C, Parkin DM. GLOBOCAN 2008 v1.2, Cancer Incidence and Mortality Worldwide: IARC Cancer Base No. 10. 2010. http://globocan.iarc.

[5] Bruni L, Albero G, Serrano B, Mena M, Gómez D et al. ICO/IARC information Centre on HPV and Cancer (HPV information Centre). Human papillomavirus and related diseases in Kenya. Summary Report. 2018. www.hpvcentre.net. Accessed 16 May 2019.

[6] World Health Organization (WHO). Comprehensive cervical Cancer control: a guide to essential practice. 2nd Edi. 2014. 25642554

[7] Ministry of Public Health and Ministry of Medical Services. Government of Kenya. National Guidelines for prevention and Management of Cervical, breast and prostate Cancers. 2012.

[8] Sankaranarayanan R. Cancer survival in Africa, Asia, the Caribbean and Central America. Introduction IARC Scientific Publications. 2011:1–5. 21675400

[9] lin QY, Sellors JW, Eder PS, ping BY, Lim JM, hui ZF (2008). A new HPV-DNA test for cervical-cancer screening in developing regions: a cross-sectional study of clinical accuracy in rural China. Lancet Oncol.

[10] Sankaranarayanan R, Nene BM, Shastri SS, Jayant K, Muwonge R, Budukh AM, et al. HPV screening for cervical Cancer in rural India. N Engl J Med. 2009. 10.1056/NEJMoa0808516 19339719

[11] World Health Organization. WHO guidelines for screening and treatment of precancerous lesions for cervical cancer prevention: WHO Press; 2013. 24716265

[12] Agurto I, Arrossi S, White S, Coffey P, Dzuba I, Bingham A (2005). Involving the community in cervical cancer prevention programs. Int J Gynecol Obstet.

[13] Arrossi S, Thouyaret L, Herrero R, Campanera A, Magdaleno A, Cuberli M, et al. Eff ect of self-collection of HPV DNA offered by community health workers at home visits on uptake of screening for cervical cancer (the EMA study): a population-based cluster-randomised trial. Lancet Glob Health. 2015;3:e85–94. 10.1016/S2214-109X(14)70354-7 25617202

[14] Sewali B, Okuyemi KS, Askhir A, Belinson J, Vogel RI, Joseph A (2015). Cervical cancer screening with clinic-based pap test versus home HPV test among Somali immigrant women in Minnesota: a pilot randomized controlled trial. Cancer Med.

[15] Crosby RA, Hagensee ME, Vanderpool R, Nelson N, Parrish A, Collins T (2015). Community-based screening for cervical cancer: A feasibility study of rural Appalachian women. Sexually Transmitted Dis.

[16] Barbee L, Kobetz E, Menard J, Cook N, Blanco J, Barton B (2010). Assessing the acceptability of self-sampling for HPV among haitian immigrant women: CBPR in action. Cancer Causes Control.

[17] De Alba I, Anton-Culver H, Hubbell FA, Ziogas A, Hess JR, Bracho A (2008). Self-sampling for human papillomavirus in a community setting: feasibility in Hispanic women. Cancer Epidemiol Prevent Biomark.

[18] Bansil, Pooja and Wittet, Scott and Lim, Jeanette L and Winkler, Jennifer L and Paul, Proma and Jeronimo J. Acceptability of self-collection sampling for HPV-DNA testing in low-resource settings: a mixed methods approach. BMC Public Health 2014;14:596. 10.1186/1471-2458-14-596 PMC4061776 24927941

[19] Ogilvie GS, Mitchell S, Sekikubo M, Biryabarema C, Byamugisha J, Jeronimo J (2013). Results of a community-based cervical cancer screening pilot project using human papillomavirus self-sampling in Kampala, Uganda. Int J Gynaecol Obstet.

[20] Rosser JI, Hamisi S, Njoroge B, Huchko MJ (2015). Barriers to cervical cancer screening in rural Kenya: perspectives from a provider survey. J Community Health.

[21] Cane J, O’Connor D, Michie S (2012). Validation of the theoretical domains framework for use in behaviour change and implementation research. Implement Sci.

[22] Michie S, van Stralen MM, West R (2011). The behaviour change wheel: a new method for characterising and designing behaviour change interventions. Implement Sci.

[23] Huchko MJ, Kahn JG, Smith JS, Hiatt RA, Cohen CR, Bukusi E. Study protocol for a cluster-randomized trial to compare human papillomavirus based cervical cancer screening in community-health campaigns versus health facilities in western Kenya. BMC Cancer. 2017;17:826. 10.1186/s12885-017-3818-z PMC5717798 29207966

[24] National AIDS and STI Control Programme (NASCOP) K. Kenya AIDS Indicator Survey (2012). Final Report. 2014.

[25] Maina WK, Kim AA, Rutherford GW, Harper M, K’Oyugi BO, Sharif S (2014). Kenya AIDS indicator surveys 2007 and 2012: Implications for public health policies for HIV prevention and treatment. Journal Acquired Immune Deficiency Syndromes (1999).

[26] Huchko MJ, Ibrahim S, Blat C, Cohen CR, Smith JS, Hiatt RA (2018). Cervical cancer screening through human papillomavirus testing in community health campaigns versus health facilities in rural western Kenya. Int J Gynaecol Obstet.

[27] Boddy CR (2016). Sample size for qualitative research. Qualitative Market Res.

[28] Braun V, Clarke V. Using thematic analysis in psychology. Qual Res Psychol. 2006.

[29] QSR International (2012). NVivo qualitative data analysis software | QSR international Pty ltd. version 10.

[30] Atkins L, Francis J, Islam R, O’Connor D, Patey A, Ivers N (2017). A guide to using the theoretical domains framework of behaviour change to investigate implementation problems. Implement Sci.

[31] Phillips CJ, Marshal AP, Chaves NJ, Jankelowitz SK, Lin IB, Loy CT (2015). Experiences of using the theoretical domains framework across diverse clinical environments: a qualitative study. J Multidiscip Healthc.

[32] Michie S, Seers K, Chandler J, Hawkes CA, Crichton N, Allen C (2005). Making psychological theory useful for implementing evidence based practice: a consensus approach. BMJ Qual Saf.

[33] Gottschlich A, Rivera-Andrade A, Grajeda E, Alvarez C, Mendoza Montano C, Meza R (2017). Acceptability of human papillomavirus self-sampling for cervical Cancer screening in an indigenous Community in Guatemala. Journal Global Oncol.

[34] Senkomago V, Saraiya M. Examining acceptability of self-collection for human papillomavirus testing among women and healthcare providers with a broader lens. J Women’s Health. 2017;26. 10.1089/jwh.2017.6384. 10.1089/jwh.2017.6384 PMC5569881 28414574

[35] Oranratanaphan S, Termrungruanglert W, Khemapech N (2014). Acceptability of self-sampling HPV testing among Thai women for cervical Cancer screening. Asian Pac J Cancer Prev.

[36] Sarai Racey C, Withrow DR, Gesink D (2013). Self-collected HPV testing improves participation in cervical Cancer screening: a systematic review and meta-analysis. Canadian J Public Health.

[37] Buchanan Lunsford N, Ragan K, Lee Smith J, Saraiya M, Aketch M (2017). Environmental and psychosocial barriers to and benefits of cervical Cancer screening in Kenya. Oncologist.

[38] Arrossi S, Ramos S, Straw C, Thouyaret L, Orellana L (2016). HPV testing: a mixed-method approach to understand why women prefer self-collection in a middle-income country. BMC Public Health.

[39] Lim JNW, Ojo AA (2017). Barriers to utilisation of cervical cancer screening in sub Sahara Africa: a systematic review. European J Cancer Care.

[40] Zachary O, Turan J, Bukusi EA, Cohen CR, Gray GEMK. “You know you are sick, why do you carry a pregnancy again?” applying the socio-ecological model to understand barriers to PMTCT service utilization in Western Kenya. J AIDS Clin Res. 2015;6. 10.4172/2155-6113.1000467 PMC4596237 26457229

[41] Tung W-C, Nguyen DHT, Tran D (2008). Applying the transtheoretical model to cervical cancer screening in Vietnamese-American women. Int Nurs Rev.

[42] Daley E, Alio A, Anstey EH, Chandler R, Dyer K, Helmy H (2011). Examining barriers to cervical cancer screening and treatment in Florida through a socio-ecological lens. J Community Health.

[43] Kivuti-Bitok LW, Pokhariyal GP, Abdul R, McDonnell G (2013). An exploration of opportunities and challenges facing cervical cancer managers in Kenya. BMC Research Notes.

[44] Hill D, Gardner G, Rassaby J (1985). Factors predisposing women to take precautions against breast and cervix Cancer. J Appl Soc Psychol.

[45] Mutyaba T, Mmiro FA, Weiderpass E (2006). Knowledge, attitudes and practices on cervical cancer screening among the medical workers of Mulago hospital, Uganda. BMC Med Educ.

[46] Morema EN, Atieli HE, Onyango RO, Omondi JH, Ouma C (2014). Determinants of cervical screening services uptake among 18-49 year old women seeking services at the Jaramogi Oginga Odinga teaching and referral hospital, Kisumu, Kenya. BMC Health Serv Res.

[47] McMullin JM, De Alba I, Chávez LR, Hubbell FA (2005). Influence of beliefs about cervical cancer etiology on pap smear use among Latina immigrants. Ethn Health.

[48] Breitkopf CR, Pearson HC, Breitkopf DM (2005). Poor knowledge regarding the pap test among low-income women undergoing routine screening. Perspectives on sexual and reproductive health.

[49] Jirojwong S, Thassri J, Skolnik M (1994). Perception of illness and the use of health care givers among cervical cancer patients at Songkla Nagarind hospital. A study in southern Thailand. Cancer Nurs.

[50] Maaita M, Barakat M (2002). Jordanian women’s attitudes towards cervical screening and cervical cancer. J Obstet Gynaecol.

[51] Rosser JI, Njoroge B, Huchko MJ (2015). Changing knowledge, attitudes, and behaviors regarding cervical cancer screening: the effects of an educational intervention in rural Kenya. Patient Educ Couns.

[52] Li M, Nyabigambo A, Navvuga P, Nuwamanya E, Nuwasiima A, Kaganda P (2017). Acceptability of cervical cancer screening using visual inspection among women attending a childhood immunization clinic in Uganda. Papillomavirus Res.

